# Perception of material appearance: A comparison between painted and rendered images

**DOI:** 10.1167/jov.21.5.16

**Published:** 2021-05-17

**Authors:** Johanna Delanoy, Ana Serrano, Belen Masia, Diego Gutierrez

**Affiliations:** 1 Universidad de Zaragoza, I3A, Zaragoza, Spain; 2 Universidad de Zaragoza, I3A, Zaragoza, Spain; 3 Max Planck Institute for Informatics, Saarbrücken, Germany

**Keywords:** material perception, depiction of material appearance

## Abstract

Painters are masters in replicating the visual appearance of materials. While the perception of material appearance is not yet fully understood, painters seem to have acquired an implicit understanding of the key visual cues that we need to accurately perceive material properties. In this study, we directly compare the perception of material properties in paintings and in renderings by collecting professional realistic paintings of rendered materials. From both type of images, we collect human judgments of material properties and compute a variety of image features that are known to reflect material properties. Our study reveals that, despite important visual differences between the two types of depiction, material properties in paintings and renderings are perceived very similarly and are linked to the same image features. This suggests that we use similar visual cues independently of the medium and that the presence of such cues is sufficient to provide a good appearance perception of the materials.

## Introduction

**Figure 1.**
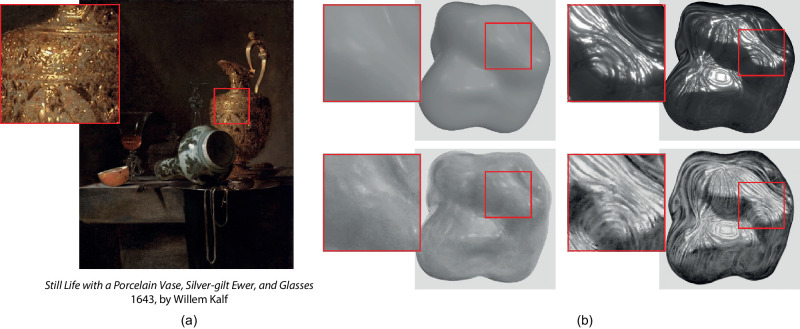
Painters do not reproduce the exact optical behavior of materials. However, they can successfully reproduce the key visual cues that lead to the perception of material appearance. In this article, we explore and analyze the perception of material properties, correlating subjective judgments with objective image features in both paintings and renderings. (a) A painting from the 17th century: The painter placed very bright spots over the surface, not necessarily aligning them with shape features, to hint the metallicity of the carafe. (b) Samples of our stimuli (top: renderings, bottom: the corresponding paintings): Paintings exhibit visual differences with the original renderings, such as differences in luminance or texture.

For centuries, painters have been able to depict the material of objects with an extreme fidelity. When looking at paintings from the 17th century, for example, the resemblance of the different metals, fabrics, or fruits is striking. Looking more precisely at some of these paintings reveals that painters do not always exactly reproduce the full optical behavior of each material but rather place some key image features that give the viewer the illusion of a specific material. For example, in the paintings shown in Figure 1a, the painter placed bright spots of paint to suggest strong metallic highlights. This suggests that painters have an implicit understanding of our perception of such materials and are able to use this knowledge to create this illusion (Cavanagh, 2005). However, the perception of material appearance is a long-standing problem that is not yet fully understood, and there is no clear consensus among vision scientists about the underlying processes it entails.

One theory suggests that our brain solves for an inverse optics problem to get the physical properties of materials and disentangle them from lighting and shape (see, e.g., the survey by Pizlo et al., 2001). However, several studies have found that varying the shape and illumination in a scene can induce drastic changes in the way we perceive materials and that these changes can be captured by specific image statistics (Marlow et al., 2012). A second theory thus suggests that we may use image statistics to recognize materials, matching them with past experience (Adelson, 2008; Fleming, 2014) or using them to encode our visual world (Fleming & Storrs, 2019). Subsequently, a variety of works have attempted to understand which image features are key to perceive specific material properties such as glossiness or metallicness (Motoyoshi et al., 2007; Anderson & Kim, 2009; Kim et al., 2012; Marlow et al., 2012; Todd & Norman 2018). The latest approaches (Fleming & Storrs, 2019) argue whether our visual system derives material perception from simple image statistics or rather use more complex highly nonlinear encodings of the visual input. The ability of painters to depict materials with only a few brush strokes suggests that they have integrated these important visual cues in order to use them in their paintings.

Studying how people perceive materials in paintings and which techniques are used by painters can thus be a means to further understand material perception. This has already brought vision scientists to look closer at paintings and link painters’ techniques with human perception (Cavanagh, 2005; Di Cicco et al., 2018; Sayim & Cavanagh, 2011; van Zuijlen et al., 2020). For example, to depict glossiness, painters usually place a lighter spot of paint aligned with the curvature of the objects (Cavanagh et al., 2008). The contrast and sharpness of this spot provide a strong cue of the perceived glossiness (Di Cicco et al., 2019). However, preceding studies have been made on already painted stimuli that (a) usually depict identifiable objects in context and (b) prevent from comparing the material perception between the painting and other renditions of the same scene. Furthermore, it does not allow to have access to the chronology of how the painting was made.

In this work, we collect professional realistic paintings of rendered materials on abstract shapes devoid of semantic meaning and directly compare the perception of material properties in the paintings and in the rendered images. In particular, we selected eight materials covering a wide appearance range and rendered them on an abstract shape previously used in various material perception studies (Bousseau et al., 2013; Vangorp et al., 2017), thus avoiding any possible bias relative to the shape and the semantics of the object. We then hired a professional artist to realistically paint the materials depicted in the renderings. We collected *subjective measures* of material appearance in these images by showing both the paintings and the renderings to participants of a perceptual study in which we asked them to rate high-level perceptual properties (e.g., *glossy*, *rough*), as well as to perform a reflectance matching task. These *subjective measures* form our basis to compare material perception in paintings and in renderings. We also extract a variety of image features (*objective measures*) that reflect material properties, such as the *highlights sharpness* or the *image contrast*. As shown in Figure 1b, the paintings and the renderings exhibit visual differences that we characterize with our image features, notably the texture of the brush strokes. Finally, we jointly analyze these subjective and objective measures in order to understand how these differences impact the perception of material properties.

Our study reveals that, despite these differences, the participants of the perceptual study perceived the materials very similarly in the two modalities. It also shows that images features in both modalities are linked to material properties in the same way: The same image features explain the same reflectance properties in both paintings and renderings. Additionally, analyzing the painting process revealed that these image features correspond to the main visual cues used by the painter. This indicates that the painter has indeed implicitly learned this knowledge about the important visual features for material perception. Our study thus suggests that, despite important differences in the style of the images, the presence of certain similar image features that were carefully reproduced by the painter allows us to perceive similar material properties.

### Material perception

A large body of work has been devoted to understand how we perceive material reflectance properties in images. Many of these works focus on the understanding of gloss and its dimensions. Pellacini et al. (2000) introduced a new reflection model based on perceived surface gloss, where variations in the dimensions of the model were perceptually uniform. Wills et al. (2009) showed that these two main dimensions driving gloss perception were related to the specular reflections and the overall brightness. Later, it was suggested that there is an additional perceptual dimension of gloss beyond these two that is related to the impression of hazy gloss (Vangorp et al., 2017). Recently, Toscani et al. (2020) have identified three dimensions for specular and diffuse reflection: gloss, lightness, and metallicity, which seem to be key for describing reflectance properties. For a review on the perception of gloss, we refer the reader to the work of Chadwick and Kentridge (2015).

Other works focus on identifying the exact image features that are responsible for the impression of gloss. It was initially found that simple histogram statistics, such as skewness, have an effect on the perception of gloss (Motoyoshi et al., 2007), but it was later demonstrated that such simple statistics are not enough to explain the perception of gloss in complex images (Kim & Anderson, 2010; Anderson & Kim, 2009). Marlow et al. (2012) showed that the perception of gloss is correlated with the coverage, the sharpness, and the contrast of highlights. However, such features are difficult to extract automatically from images and require human judgment to be studied, introducing possible bias. We take inspiration from these works by extracting image features that are linked to material perception from both the paintings and renderings. In particular, we use both local features, such as the sharpness and contrast of the highlights, and global features, such as the contrast and the luminance of the image.

These simple highlights statistics are not sufficient to explain the perception of gloss; they also need to be properly placed on the surface, close to the most luminous parts of the diffuse shading and elongated according to the surface curvature (Kim et al., 2011; Marlow et al., 2011). The lowlights, the darker specular reflections, are also key to the perception of gloss and need as well to be properly aligned with the shape (Kim et al., 2012). However, apparent image features do not solely depend on the reflectance properties of the material but also on the shape and illumination, which has been shown to play a major role in the perception of gloss (Olkkonen & Brainard, 2011). Notably, it was shown that the estimation of gloss is more accurate under natural illuminations (Fleming et al., 2003), which led us to use a natural illumination in our study.

Fewer works have been devoted to the study of other dimensions of material perception, such as distinguishing between different types of materials. Filip and Kolafová (2019) recently performed an analysis on perceptual attributes in real-world materials in which they assessed several tactile and visual attributes and evaluated the relationship between such attributes and different material categories. They also compared the perception of such material attributes when viewing a computer graphics rendering to those when viewing a physical sample of the same material (Filip et al., 2018). They found that participants evaluated perceptual attributes more consistently between these two modalities for familiar materials than for materials with random structures. Although several studies have investigated how we perceive different materials (e.g., wood, stone, or fabric) on pictures of textured objects, very few have investigated this question based solely on reflectance properties. Todd and Norman (2018) focused on the perception of metal and showed that the feeling of metal is due to the predominance of specular reflections over diffuse ones: Metals exhibit almost no diffuse reflections but strong specular ones, causing a large coverage of reflections. We incorporate into our study both metallic and nonmetallic materials in order to cover a wide range of materials. Although we do not focus on understanding the distinction between these two types of materials, our results suggest that the same image features can be used similarly for both types for materials in order to explain high-level perceptual properties.

All these works show that our perception of material properties is an intricate mixture of various image cues. A recent work suggests that material perception might arise from the nonlinear encoding of our world that our brain performs (Fleming & Storrs, 2019). The authors build a parallelism between this encoding and the encoding performed by deep neural networks, suggesting that our perception of a material might be driven by complex nonlinear statistics similar to the ones extracted by neural networks. Related to this, Lagunas et al. (2019) showed that a deep neural network could be trained to learn a notion of material appearance similarity from images. In order to compare the perception of material properties in paintings and renderings, we use simple image features that are easy to compare and to relate to the painting process. Although they may not fully represent the complexity of image features that humans use, we show that they are enough to explain an important part of the perception of high-level material properties.

### Perception in artistic depictions

It is well known that painters use shortcuts and even an “alternative physics” when depicting reality (Cavanagh, 2005), usually in a way that is not obviously noticeable for human eyes. Analyzing these shortcuts can thus help us to understand how we perceive our world. These studies cover various subjects such as our perception of transparent objects (Sayim & Cavanagh, 2011) or of reflections (Cavanagh et al., 2008). For example, it was shown that we are not sensitive to inconsistencies in illumination in a scene (Ostrovsky et al., 2005) and that many paintings exhibit such inconsistencies in favor of visual effect (Cavanagh, 2005). In this work, we focus on the depiction of materials and investigate which image features seem to be key to reproduce for the painter and if they align with previous findings about material perception.

Also focusing on material depiction, Di Cicco et al. (2019) analyzed grapes paintings from the 17th century and found out that the clues identified by Marlow et al. (2012) were a good predictor of the gloss of grapes in the paintings. They thus suggest that material perception depends on similar image features in paintings and natural images. Cavanagh et al. (2008) showed that it is sufficient for highlights to be lighter than the rest of the surface and deformed according to the curvature of the object to look real. These works have thus identified a few image features that may be used implicitly by painters to convey object appearance. However, they are based on already painted artworks that forbid us to directly compare the perception of material properties in paintings and in natural images.

In a recent study (Di Cicco et al., 2018, 2020), a painter reproduced grapes depictions from the 17th century, following both analysis of the original painting and recipes described in a 17th-century art treatise, in order to analyze the influence of each layer on the perception of the grapes. However, this study does not investigate the perception of general material properties but rather the perception of specific properties of the grapes with the goal to understand if the recipes described in the art treatise were perceptually relevant. For the case of complex textured materials, van Zuijlen et al. (2020) studied the perception of various materials in paintings from the 17th-century. They found out that the properties associated with each class of material is very similar to the ones reported with natural images. This, again, suggests that our perception of material is not dependent on the medium.

In our work, we seek to learn about visual features and their influence on material appearance perception by gathering data from *coupled* depictions of materials: a rendered image and a painting for each material sample. This direct comparison between both renditions is, to our knowledge, novel. Similar in spirit to this, the study from Bousseau et al. (2013) studied the influence of various non photorealistic rendering (NPR) algorithms on the perception of gloss. In contrast to NPR automatic algorithms, in this work, we focus on actual paintings that incorporate human knowledge and artistic experience into the “stylization” process.

## Stimuli generation: Renderings and paintings

As our material samples to be painted, we selected eight materials from the MERL data set (Matusik et al., 2003), which contains 100 measured materials from the real world, stored as BRDFs (bidirectional reflectance distribution functions, which define how light is reflected off a surface for every incoming light direction). There are a number of analytical BRDF models that aim at reproducing the appearance of real-world materials, but they usually are not able to fully reproduce the richness of visual effects that real-world materials can exhibit. In contrast to analytical BRDF models, so-called measured BRDFs model the appearance of a surface as large tables storing the amount of light reflected for every pair of incoming and outgoing directions. Thus, measured BRDFs allow having a fully realistic appearance, at the cost of not providing an easy control over the properties of the material.

Our selection contains eight materials that span a large variety of appearances; three of them correspond to metallic materials with a dominant specular component ($M_{1..3}$) while the others ($P_{1..5}$) have a dominant diffuse component. For simplicity, we order the materials in each group by increasing glossiness.

These materials were rendered under a fixed viewpoint on the blob shape used in previous perceptual studies (Vangorp et al. 2007). This shape is simple enough to be drawn easily by the painter while being more effective than a sphere in conveying the right material properties. We used a natural illumination since this favors material perception (Fleming et al., 2003) and chose the *Eucalyptus* environment (Debevec, 1998). This illumination contains numerous bright areas alternating with darker ones, allowing for interesting reflection effects to be painted over the shape rather than only a few bright points.

The renderings were all made using the Mitsuba renderer (Wenzel, 2010) and tone-mapped using Mantiuk et al.'s operator (Mantiuk et al., 2008), with the predefined *lcd* display, and color saturation and contrast enhancement set to 1. The renderings were then converted to grayscale by using the luminance channel of the Lab space to eliminate the effect of color; background information was masked.

We then recruited a professional artist specialized in hyperrealist painting^1^ to paint the material samples on an empty background. The exact goal given to the painter was to “paint the same material that is presented in the rendering,” thus concentrating on giving the right “material impression.” The renderings were shown on an iPad and kept visible during the entire painting process. The paintings were made using acrylic paint on cardboards of size 18 × 24 cm. The paintings were done over five sessions of 4 hr each; all of them were recorded on video. We then photographed the paintings and treated the photographs to correct for perspective error and mask out the paper background. The paintings were photographed at a resolution of 1,910 × 1,910 pixels and then downscaled to a resolution of 500 × 500 pixels, the same size of the renderings, allowing us to eliminate photography noise while keeping the texture of the paintings. All images (paintings and renderings) were placed on a gray background with luminance L=0.88, which is equivalent to the luminance of the cardboard.

**Figure 2.**
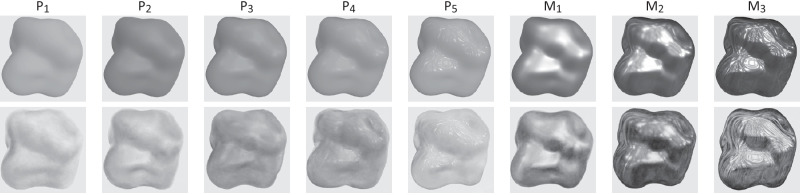
Stimuli used in our perceptual study: First line shows the renderings; second line shows the paintings of the eight materials. $P_{1}$ to $P_{5}$ have a dominant diffuse component; $M_{1}$ to $M_{3}$ correspond to metallic materials with a dominant specular component. Both are ordered by increasing glossiness. Higher-resolution images can be found in the Appendix C.

The renderings and the corresponding paintings are shown in Figure 2. While trying to match the reflectance properties of the material, the painter did not attempt to match the brightness of the renderings. He reported afterward that he did not consider this an important parameter to convey the material properties. In the perceptual study (see Subjective measures: User-study methodology), users globally perceived the material properties similarly between the paintings and the renderings, showing that the painter succeeded in reproducing the material appearance of the rendered materials.

### Painting process

**Figure 3.**
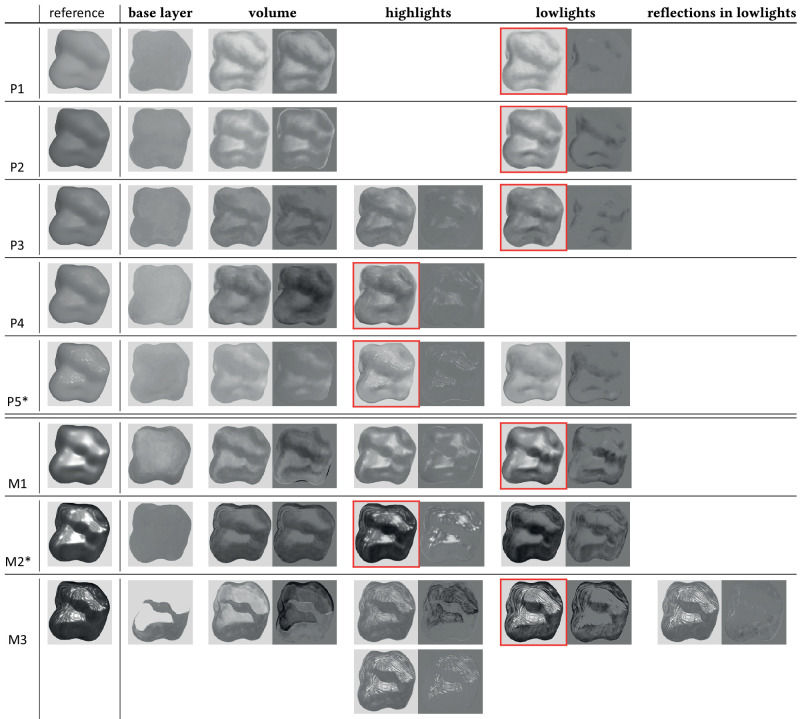
Main steps of the different paintings, classified by the main phases of material representation. The reference image is shown in the first column. In each column, the left image shows the painting while the right image shows the extracted layer of paint (difference with the previous painting). For the paintings marked with a star (P5 and M2), the lowlights were painted before the highlights. The red outline indicates the final painting.

In order to understand how the painter built his representation of the material and which features seem to be key for him, we analyze the painting process of each image. Using the recording of the painting sessions, we decompose the paintings in steps, corresponding to changes in the color that the painter was currently using. This allows us to separate the phases of painting the shadows from painting the highlights, for example. The choice of the decomposition is also guided by the explanations given by the artist during the session. We align the keyframes when needed by registering points placed on salient features of the painted shape and canvas through perspective transformation. We extract the different layers by subtracting each step from the next one. For visualization purposes, we then add this difference to a mid-gray layer, allowing us to clearly see the dark or bright brushes of the layer. The different steps are shown in Figure 3, where each column shows the painting at a given step (left image) as well as the extracted layer (right image).

These steps can be categorized in four main phases, although each one is not necessarily represented in each painting, and their order can vary:

(1) Creating a first *base layer*, almost completely constant in color.
(2) Building the *volume*: The painter builds the first light contrast between luminous parts of the shape and shadow parts, either by lightening or darkening the base layer. This builds the main volume of the shape. At this stage, the contrast of all paintings is similar, showing that the material does not play a role yet.
(3) Adding *highlights*: The painter uses diluted white color to add the highlights. Globally, the highlight layer has more contrast for the metallic materials, with the use of less diluted paint. The paintings of $P_{1,2}$ do not have a separated highlights layer because the materials are mostly diffuse.
(4) Adding *lowlights*: The painter reinforces some shadows with black or almost black paint. For nonmetallic materials, this consists of only adding a few shadows to adjust the volume. For metals, the painter added lowlights in almost all the shadowed part of the shape, producing much darker and complex shadows than that for the nonmetals. For the most specular metal $M_{3}$, the painter added a layer of reflections in the dark area before reinforcing the shadows (shown in the last column of Figure 3).

This process reveals the important visual clues that the painter tried to reproduce:

• The sharpness, contrast, and placement of the highlights
• The intensity of lowlights (or the contrast between diffuse shadows and lit areas)
• The potential reflections in shadow regions

While the two first ones are influenced by how glossy the material is perceived, the two last ones are responsible for the metallic impression. These features have previously been shown to be key to the perception of several reflectance properties such as glossiness or metallicness, suggesting that the painter indeed knows the key features to material perception.

These clues differ substantially according to the material to depict but provide insights as to what features are most relevant in depicting the material. We will look into those in Objective measures: Image features.

## Subjective measures: User-study methodology

We aim to compare the perception of materials on two different depictions: realistic renderings and paintings of these renderings, provided by a professional artist. For this purpose, we carried out two different experiments. For each material and depiction, in the first experiment, we asked the participants to rate a series of high-level perceptual attributes, and in the second experiment, we asked them to perform a reflectance matching task, in which they had to adjust the parameters of a BRDF model to match a reference image (rendering or painting). While the matching task yielded a more accurate view of the low-level perception of the material, the high-level perceptual questions allowed us to measure how participants perceived material traits overall, without a direct image comparison. In this section, we first describe common aspects of the experimental procedure for both experiments, and then we describe specific aspects of each experiment.

### Common experimental procedure

#### 

##### Stimuli

The stimuli consist of images showing the two different depictions (renderings and paintings) of eight materials, generated as described in Stimuli generation: Renderings and paintings. All the stimuli are shown in Figure 2.

##### Procedure

**Figure 4.**
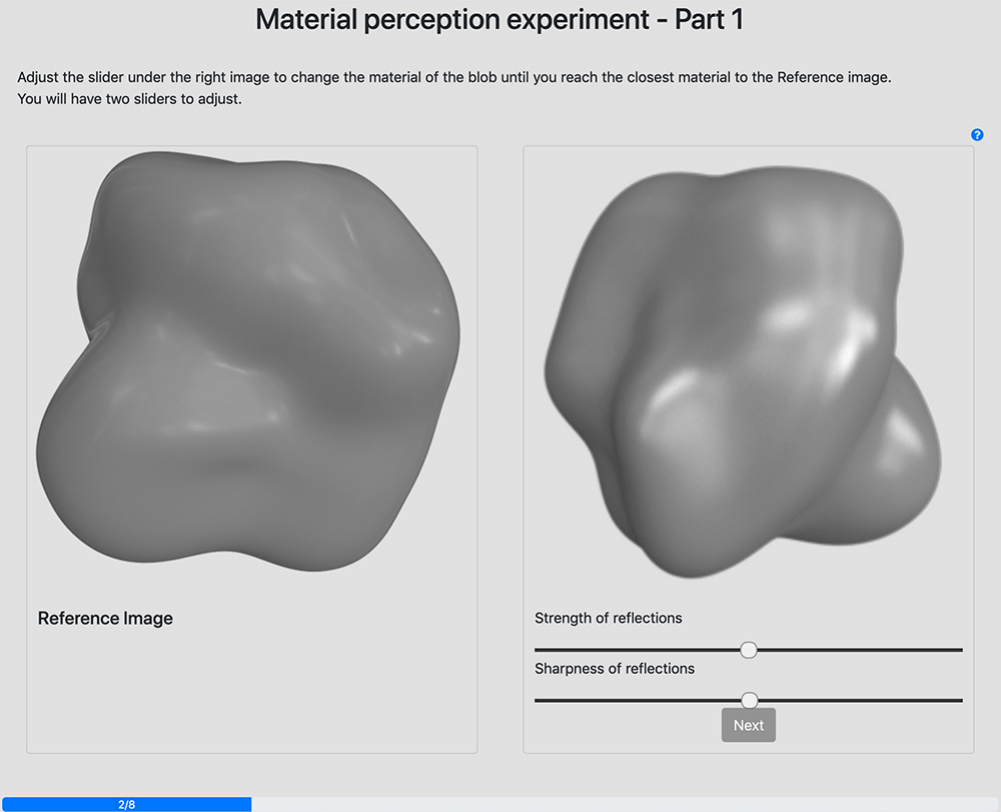
Screenshot of the experiment during the matching task. The reference image (painting or rendering) is always shown on the left half of the screen while the right half contains the questionnaire during the rating task or the test image and the two sliders during the matching task.

We implemented our experiment in a web browser. During the test, participants were shown one reference image (a rendering or a painting) at a time. This reference image was shown on the left half of the screen, while the right half displayed the questionnaire for either the rating or the matching task (a screenshot of the experiment during the matching task is shown in Figure 4). Both experiments were divided in two sessions: one with the renderings, one with the paintings. In each session, the order of the stimuli was randomized, as well as the order of the two sessions. In order to avoid remembering the materials from one session to another, participants were asked to take at least a 15-min break between the two sessions. In the explanation of the painting session, participants were told that they were looking at paintings.

##### Participants

Sixteen participants (average age 26.5 years, σ = 5.13) took part in the study. From this participant pool, 15 had some experience in computer graphics, and 13 had some art experience.

##### Training

For both experiments, participants first had to complete a training session containing two stimuli. They were shown explanations of the different tasks, as well as descriptions and examples of the different attributes they had to rate during the perceptual questions. These explanations were also available on demand during the whole duration of the experiment. The training stimuli consisted, of BRDF renderings using the same analytic model that was used during the matching task (see Matching task). The parameters of the model for the training examples were chosen to depict extreme values of the different perceptual properties to rate, such that they would clearly convey the meaning of each property.

### Attribute rating task

Participants were first asked to classify the material in one of the following categories: “Metal,” “Plastic,” “Fabric,” “Other.” This classification task gives a high-level view of understanding the material by the participants. We then asked participants to rate five perceptual properties on a 5-point Likert scale, following previous works (Serrano et al., 2016; Zell et al., 2015; Du et al., 2013). In particular, our questionnaire contains three common high-level properties that cover various aspects of material perception: *glossiness*, *brightness*, and *roughness*, as well as two lower-level properties that relate to the specularity of the material: *sharpness of reflections* and *strength of reflections*.

### Matching task

For the second experiment, we asked participants to match the reflectance of a test image to the reflectance of a reference image (one of our stimuli, painting or rendering). The test stimuli were rendered using the isotropic Ward model (Ward, 1992), which represents the reflectance as the sum of a diffuse and a specular component. This model has the advantage of being represented with only three parameters that can easily be linked to the perception of the gloss of the surface (Pellacini et al., 2000): $\rho_{D}$ (diffuse reflectance), $\rho_{S}$ (energy of the specular component), and α (spread of the specular lobe). In a pilot study, we found that matching the three parameters of the Ward model was very difficult, even for computer graphics experts. Similarly to related studies in material perception (Fleming et al., 2003; Bousseau et al., 2013; Vangorp et al., 2017), we thus fixed the diffuse parameter ($\rho_{D}$), which determines the albedo of the surface.

In order to find the fixed value for the diffuse parameter that better approximated our stimuli, four experienced users (two of the authors and two volunteer participants) matched the full Ward model to each stimulus. The average variances in their answers for the matching task were low: 0.1 for the paintings and 0.01 for the renderings (recall that in the Ward model, each parameter can take values between 0 and 1); therefore, we averaged the answers to get a reference value for $\rho_{D}$. Participants were thus asked to match only the two parameters of the specular component: α (called *sharpness of reflections* in the experiment) and $\rho_{S}$ (called *strength of the reflections*). The sliders were named such that they refer to properties rated by the participants in the perceptual ratings, making it easier for them to understand their meaning.

The steps follow the perceptual scale defined in the the work of Pellacini et al. (2000). Specifically, values for α ranged from 0.001 to 0.239 with steps of 0.0125. Although it was reported that the surface becomes diffuse for α>0.2 (Ward, 1992), we slightly extend, that range to avoid ceiling effects as reported by Bousseau et al. (2013) and chose a similar range to theirs. For the strength of reflections ($\rho_{S}$), the contrast as defined in Pellacini et al. (2000) ranged from 0 to 0.8 with steps of 0.02. The corresponding range for $\rho_{S}$ was computed with a fixed diffuse component of 0.2 such that the possible values for the specular parameter were the same for all stimuli, while ensuring that $(\rho_{S}+\rho_{D})<1$ to avoid reflecting more light than the incoming light. In the rest of this article, we report answers for $\rho_{S}$ using the same non contrast scale. For both sliders, the scale was sampled with half steps for the first 20% of the scale as our pilot experiments show that the steps were too big for fine adjustments at the beginning of the range.

The test images were then rendered using the same blob shape with a different viewpoint and same illumination as the reference image. Using a different viewpoint avoids pixel-to-pixel comparison between the two images.

Participants were shown the test image on the right with the two sliders that control the sharpness and strength of reflections under it (Figure 4). Participants were asked to move the slider such that the material in the test image appeared as similar as possible to the material of the reference image. After validating the task, participants were asked to score how satisfied they felt with the matching on a 5-point scale bounded by *extremely unsatisfied* and *extremely satisfied*.

## Objective measures: Image features

In order to analyze the answers of the perceptual study, we first seek to identify which important image features may help to convey the appearance of the material and which ones are also reproduced in the paintings. We thus compute a variety of features that are either known to be linked to material appearance, such as the *highlights sharpness*, or that are specific to the paintings, such as the *local perturbation* in the images.

**Figure 5.**
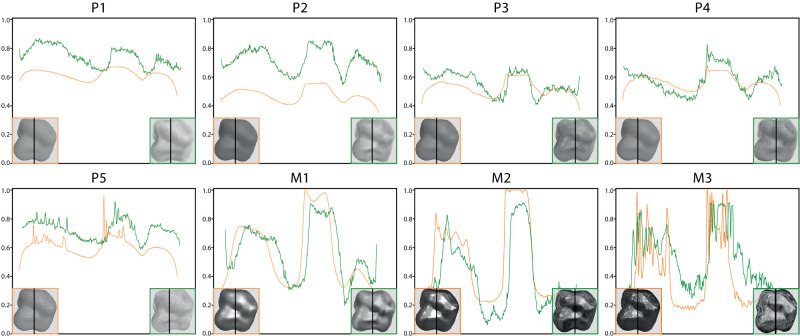
Profiles extracted along the vertical black line depicted in the images, which covers both the most luminous and darker parts of the shape. The orange line is the profile extracted from the renderings, while the green one is extracted from the paintings.

**Figure 6.**
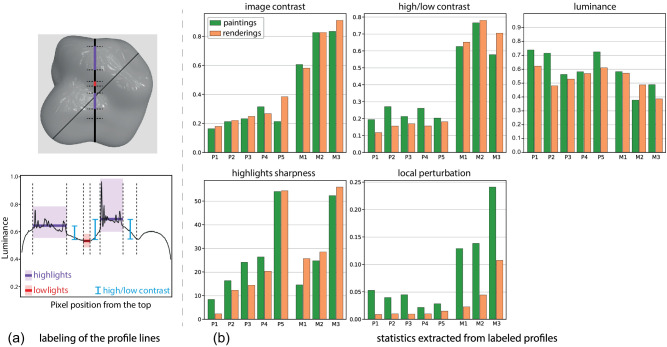
(a) Top: The rendering of *P*_5_ with the two lines (in black) that we use in our analysis. Bottom: The profile extracted from the bold vertical profile. We cut the profile at the dashed lines and label the different zones as the “highlights zones” (in purple) and “lowlights zones” (in red). The difference in average luminance between these zones (in blue) is our *high/low contrast*. (b) Image features that we extract from the luminance profiles, on paintings and renderings. Each measure is pooled (maximum or average) over the different profiles.

Following the work of Di Cicco et al. (2019), we extract the luminance profiles from both types of images (renderings and paintings) in order to identify the most prominent features. We choose two lines (one vertical, one diagonal) that run through the most luminous and sharp highlights as well as through the shadows of the shape. These positions of the lines allow us to compare both the luminance difference between the most luminous and darkest parts, as well as the contrast and sharpness of highlights. We average the profiles over 3-pixel-wide lines. The profiles extracted from the vertical line are shown in Figure 5, where the orange profiles correspond to the renderings, while the green ones correspond to the paintings. Looking at the profiles allow us to visually appreciate the differences between the two modalities, of images and between the different materials, such as differences in luminance (vertical translations), in contrast (vertical amplitude), or in the shape of highlights. The second line (diagonal one) that we use to compute our features is shown in Figure 6 (top left).

In order to extract the features from the profiles, we manually cut and label them into a “highlights zone” (shown in purple in Figure 6, left), a “lowlights zone” (shown in red), and transitions in between. This labeling is made by looking simultaneously at the luminance profiles and the input image. From these labeled profiles, we extract five features:

• *Image contrast*: Michelson contrast (Michelson, 1927) obtained as $\left(I_{max}-I_{min}\right)/\left(I_{max}+I_{min}\right)$.
• *High/low contrast*: difference between the average luminance in the highlights and lowlights, shown as the blue lines in Figure 6 (left). Contrary to the *image contrast*, this measure does not account for the shape of the highlights.
• *Luminance*: average luminance value.
• *Highlights sharpness*: width of the highlights transition, similar to Di Cicco et al. (2019). This is measured as the amplitude of the highlights divided by the maximum derivative of the highlight peaks. We further explain the computation of this feature in Appendix A.
• *Local perturbation*: standard deviation of the first derivative from a smooth version of the profile obtained with a bilateral filter. We do not take into account the highlights zone in this measure to avoid measuring the strength of the highlights. This measures small-scale perturbations in the image, such as brush strokes in the paintings.

For the features that relate to a luminance amplitude (*image contrast* and *high/low contrast*), we keep the maximum value over the different zones and profile lines and average the rest. The final extracted features are shown in Figure 6.

The image features span a large range of values, which endorses our selection of materials since we chose them so that they would cover a wide range of appearances. Only the *luminance* spans a short range of values since we purposely chose materials with a similar luminance in order to allow easier comparison between them.

The image features also reflect our organization of the materials. Both the *highlights sharpness* and the *image contrast* are in accordance with the ordering of the materials, increasing from $P_{1}$ to $P_{5}$ and from $M_{1}$ to $M_{3}$ in both types of images. The metallic materials ($M_{1..3}$) have much higher contrasts (both *high/low contrast* and *image contrast*) than the nonmetallic ones ($P_{1..5}$), in accordance with their dominant specular component.

Comparing the image features and the image profiles between the paintings and the renderings allows us to observe three major differences between both modalities:

• For $P_{1..4}$, both the *high/low contrast* and *highlights sharpness* are higher in the paintings, and the transition from shadow to light appears steeper in the profiles. The paintings of these nonmetal materials thus seem to exhibit exaggerated light effects. Interestingly, the painter stated that the nonmetals, particularly the most diffuse ones, were the hardest to reproduce due to the subtlety of the light effects on these surfaces. This difficulty to reproduce subtle effects could explain the image differences between the paintings and the renderings for these materials.
• The *local perturbation* is much higher (between two and five times higher) in the paintings than in the renderings. This is due to the texture of the brush strokes that is clearly visible in the paintings. This is one of the most important visual differences between the renderings and the paintings.
• The *luminance* exhibits important differences between the paintings and the renderings and particularly higher values in the paintings of $P_{1,2,5}$. Interviewing the painter reveals that he on purpose did not try to match the luminance of the reference image because, according to him, it is not what is responsible for the impression of the material since it is equivalent to showing the same material under a more intense light.

**Figure 7.**
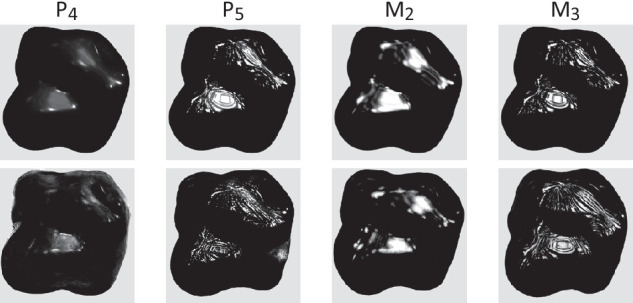
For a subset of the materials, highlights extracted from the renderings (top row) and from the paintings (second row). Please refer to the text for details.

In the remainder of the article, we will seek to understand how these differences have impacted the perception of material properties in the perceptual study. Note that material appearance perception may not be fully explained by the set of image features included here. For example, the placement and shape of highlights have been shown to be relevant for the impression of gloss (Kim et al., 2011; Marlow et al., 2011) but cannot be easily measured or quantitatively compared in our set of stimuli. We did assess qualitatively whether the position and orientation of highlights were consistent between paintings and renderings by extracting the highlights of each depiction for the materials that exhibited the clearest highlights ($P_{4,5}$ and $M_{2,3}$). More precisely, we selected the most luminous pixels of the images by manually setting the minimum and maximum luminance values (changing the levels). These highlights are shown in Figure 7. Because the painter did not seek to make an exact copy of the renderings, the exact shape, number, and placement of the highlights are not identical between the two modalities, but the highlights follow the same global direction and orientation, are placed on the same area of the shape, and reflect a similar environment.

## How do image features explain perceptual ratings?

We seek to identify which of these image features are the most important to convey material appearance and can thus be used to explain users’ answers. We first analyze the correlations between users’ answers and image features before exploring the use of these image features as simple predictors of high-level attributes.

### Correlations between image features and perceptual ratings

**Figure 8.**
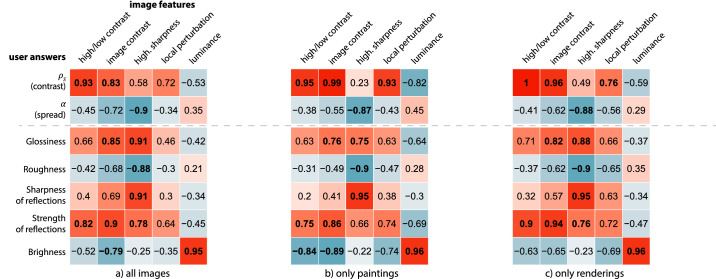
Correlations between our image features and the users’ answers for (a) all the images, (b) only the paintings, and (c) only the renderings. The color scale indicates the strength and direction of the correlations (from blue to red). The most significant correlations (r>0.75) and *p*-value (p<0.001) are indicated in bold.

We seek to understand how perceptual ratings are linked to our image features and which image features better explain high-level attributes and matched parameters. We thus look for high correlations between such answers and our computed image features, which would validate the expressiveness of such features. In Figure 8, we show the Pearson correlations between the image features and the users' answers, pooling together all the images, then considering paintings and renderings separately. A blue color indicates a negative correlation, while red indicates a positive correlation. Saturation indicates the strength of such correlation. As shown in this figure, painting and rendering modalities share common patterns of correlations; therefore, we focus mainly on describing these common behaviors in the pooled results for all images and highlight the main differences between paintings and renderings when present.

The matched contrast $\rho_{S}$ and the *strength of reflections* are highly correlated with both the *high/low contrast* (r=0.93 and r=0.82, respectively) and the *image contrast* (r=0.83 and r=0.9, respectively), showing that the contrast in the image is a good cue to judge the intensity of reflections. However, while the contrast $\rho_{S}$ seems to be only influenced by the contrast between shadowed and lit areas (higher correlation with the *high/low contrast*), the *strength of reflections* is also linked to the sharpness of the highlights (higher correlation with *image contrast* and high correlation with the *highlights sharpness*). This shows that users were consistently biased by the appearance of highlights when judging the strength of reflections without visual reference (in the rating task), overestimating it when sharp highlights were visible.

The *local perturbation* is also highly correlated with the matched contrast $\rho_{S}$, particularly when taking renderings and paintings separately (r=0.93 and r=0.76, respectively). This image feature captures not only the texture of the brush strokes but also the reflections in the lowlights zones, which correlates with the strength of reflections. This correlation is particularly strong for the paintings due to the fact that reflections in the dark areas are exaggerated in the paintings of the metallic materials.

In accordance with previous studies that have shown that glossiness depends on both the contrast and the sharpness of highlights (Pellacini et al., 2000; Marlow & Anderson, 2013; Di Cicco et al., 2019), the *glossiness* attribute is highly correlated with both the *image contrast* (r=0.85) and the *highlights sharpness* (r=0.91).

Finally, all the attributes that relate to the sharpness of the highlights (α, *roughness*, *sharpness of reflections*) are highly correlated with the corresponding image features (*highlights sharpness*, r>0.9), and the *brightness* attribute is fully explained by the *luminance* of the images (r=0.96). This shows that our image features capture well the visual features that account for these attributes.

A major difference between the paintings and the renderings lies in the correlations between the *brightness* or *luminance* and all the other attributes and features. These correlations are higher in the paintings than in the renderings and may reflect a bias in the paintings where all nonmetallic materials were painted brighter.

### Using image features as predictors of high-level attributes

The high correlations that we observe between the image features and the users’ answers suggest that we can use certain image features to predict some perceptual properties of the materials. By fitting a linear model to each user's answers, we can better understand which image features are key to each perceptual attribute and compare their effect in the renderings and in the paintings. In this section, we focus on users’ answers to the attribute rating task since we are interested in users’ perceptual ratings of material appearance. Fittings for the answers to the matching task (parameters of the Ward model) can be found in Appendix B.2.

Since some of the image features are highly correlated, fitting a multivariate linear model containing all the image features would not be informative. In order to select only the smallest number of appropriate features for each user attribute, we use a forward selection method: We successively add the attribute with the lowest *p*-value if it is statistically significant (p<0.05).

**Figure 9.**
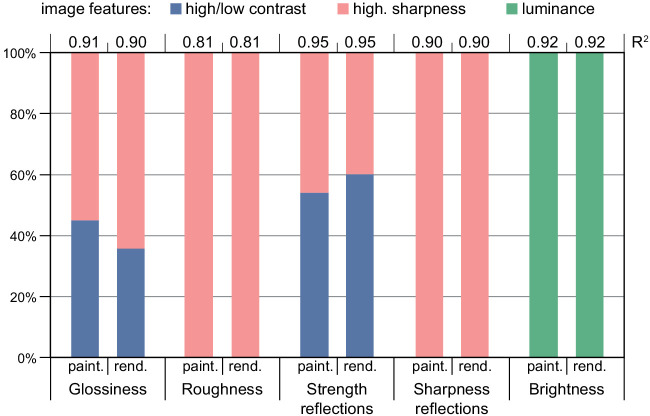
Coefficients in the linear models that predict each user's answers of the rating task. For each variable, the left bar shows the coefficients for the model fitted on the paintings while the right-most one shows the coefficients of the model fitted in renderings. The regression score for each model is displayed on top of each bar.

We compute the linear coefficients of each variable in the models and show their relative importance in Figure 9. This allows us to compare the importance of each feature when predicting a given attribute. The coefficients confirm what we observed in the correlations. The attributes *glossiness* and *strength of reflections* are predicted by both the *global contrast* and the *highlights sharpness*, the attributes that relate to the sharpness of highlights (*roughness*, *sharpness of reflections*) are predicted by the *highlights sharpness*, and the *brightness* attribute is predicted by the *luminance*. Interestingly, for the majority of the predicted variables, the importance of each image feature is very similar between the two models, fitted for paintings and renderings, respectively.

On top of the linear coefficients in Figure 9, we also show the regression score ($R^{2}$) for each of the models. Our models fit all the high-level perceptual attributes with a score over 0.9, except for the *roughness* attribute ($R^{2}=0.81$), which seems harder to explain by using only the *highlights sharpness*. This suggests that the perception of material properties in our experiment was mainly guided by simple image features that are present in both paintings and renderings. Furthermore, the scores are almost identical for the two models, suggesting again that the same image features are linked in the same way to perceptual properties of materials in both modalities.

Our analysis suggests that perceptual properties of materials are strongly linked to a few image features in both modalities. These image features can thus be used to better understand the answers of the perceptual study.

## Statistical analysis of users’ answers

In this section, we analyze in detail the answers from the perceptual study in relation to our selected image features. We seek to find if differences in users’ answers between paintings and renderings are linked to actual differences in the stimuli but also if the important visual differences that we observed between paintings and renderings had an impact on the perception of material appearance.

As a preliminary analysis, we first study the correlations between the answers in our two experiments, both interparticipants and interattributes. The main conclusion of this analysis is that in general, participants were able to consistently judge material appearance for renderings and paintings similarly well in the two tasks (rating and matching). Participants were globally consistent among each other, especially in the matching task, but some attributes were more prone to personal interpretation (*brightness* and *roughness*) and exhibited less consistency. Additionally, participants were generally less consistent when judging paintings than when judging renderings. This analysis can be found in Appendix B.1 (Figure 13).

We then conduct a statistical analysis of these answers by analyzing which factors have a significant influence on the results of both task. The factors (independent variables) we include are depiction ={renderings,paintings}, material $=\{M_{1..3},\;\;P_{1..5}\}$, and the interaction between depiction and material.

We cannot assume that our observations are independent, since each user is measured several times under different conditions; therefore, we model the potential effect of each subject as a random effect. Additionally, none of our measurements (dependent factors) are normally distributed (p<0.05 for the Shapiro-Wilk test). Therefore, in order to quantify the effect of our factors on each of our measurements, we use a generalized linear mixed model with a gamma distribution (unless stated otherwise), which is well suited for scale responses with positive values like ours. In all our tests, we fix a significance *p*-value of 0.05. Finally, for the factors that present a significant influence, we further perform pairwise comparisons when necessary through estimated marginal means (emmeans) with Bonferroni correction for multiple comparisons.

**Figure 10.**
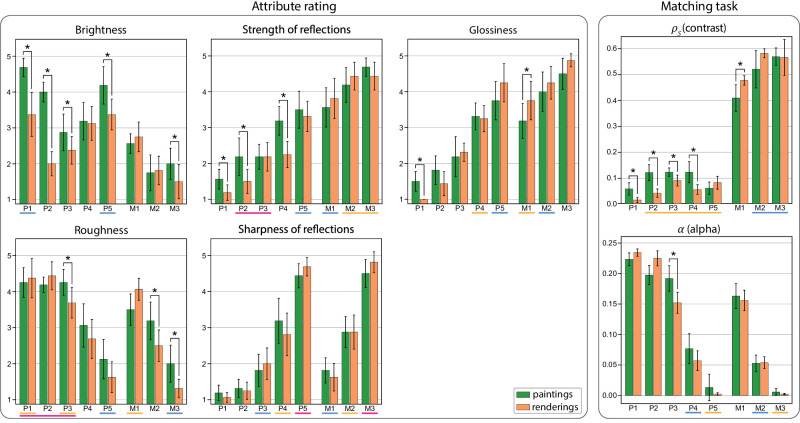
Mean rated attributes and matched values for each of the different materials according to the depiction. Error bars correspond to a 95% confidence interval. Materials underlined with the same color on the x-axis correspond to statistically indistinguishable groups. Stars indicate significance for the comparison between paintings and renderings for each material.

**Figure 13.**
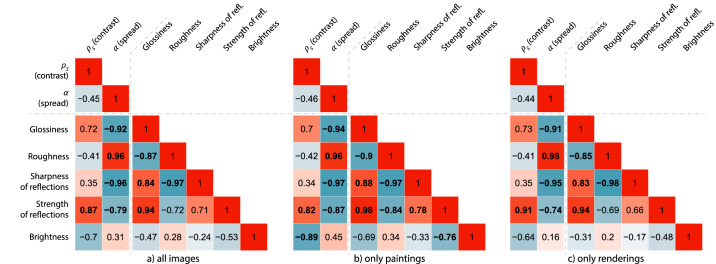
Correlations between the user answers for (a) all the images, (b) only the paintings, and (c) only the renderings. The color scale indicates the strength and direction of the correlations (from blue to red). The most significant correlations (r>0.75 and p<0.001) are indicated in bold.

For the high-level attribute rating, the dependent variables that we analyze are the attributes *glossiness*, *brightness*, *roughness*, *strength of reflections*, *sharpness of reflections*, and *time* taken to answer the questionnaire. For the matching task, the dependent variables that we analyze are the final matched values of the model ($\rho_{S}$ and α), the *satisfaction* of the user with the matching, and *time* for completing the task. The answers from the perceptual study can be seen in Figure 10, and the results of the analysis are described in the next subsections. We provide additional results, such as the results of the classification task, the time to complete the tasks, and the impact of demographics, in Appendix B.

### Influence of material

As expected, we have found a significant effect of the material in every measured attribute and matched parameter (*p* < 0.001). This is an indication that both for the renderings and paintings, participants are perceiving the different stimuli as belonging to different materials.

Similarly to the image features, the users’ answers reflect our choice and ordering of materials, both for the paintings and the renderings. In the high-level attribute rating task, materials from $M_{1}$ to $M_{3}$ and from $P_{1}$ to $P_{5}$ present increasing values for *glossiness*, *strength of reflections*, and *sharpness of reflections*, as well as decreasing *roughness* values, as can be seen in Figure 10 (left). In the matching task, the spread of the specular reflections α follows a very similar scheme, decreasing from $M_{1}$ to $M_{3}$ and from $P_{1}$ to $P_{5}$ (Figure 10, right). Finally, metallic materials ($M_{1..3}$) were matched with a much higher contrast $\rho_{S}$ (four times higher on average) than nonmetallic materials ($P_{1..5}$) and were also rated as with slightly more *glossiness* and higher *strength of reflections*. This indicates that the different depictions are successful in transmitting the different material attributes.

### Influence of depiction and interactions with material

Most attributes were, in general, well matched between paintings and renderings, indicating that material properties were perceived very similarly between the two modalities. There are three notable exceptions to this, and in the following, we look at whether differences in our selected image features can explain them or if other factors are involved. In Figure 10, we indicate significant effects of the interactions of depiction and material (*p* < 0.001) with an asterisk.

#### Differences between paintings and renderings for $P_{1..4}$

We observed a large effect size for the significant differences in ratings for the nonmetals $P_{1..4}$. The paintings of these materials were rated with significantly higher *strength of reflections* (except for $P_{3}$) and were matched with higher-contrast $\rho_{S}$ than the renderings. The most diffuse ones ($P_{1}$ and $P_{2}$) were also rated as having more *glossiness* in paintings.

This corresponds with the materials that exhibit higher *high/low contrast* and *highlights sharpness*. The painting of $P_{3}$ exhibits less difference in *high/low contrast* than the other materials, which can explain why it was not rated with significantly higher *strength of reflections*. These two measures (*high/low contrast* and *highlights sharpness*) jointly explain well the perceived strength of reflections and glossiness: We found high correlations between difference in these answers (*glossiness*, *strength of reflections*, and $\rho_{S}$) and the difference in *high/low contrast* (around r=0.8) and *highlights sharpness* (around r=0.75). This confirms that the difference in perception ratings can be explained by actual differences in the stimuli.

In summary, for these particular materials ($P_{1..4}$), not only the visual cues are not same, but the final perceived appearance is significantly different between the renderings and the paintings, indicating that the material depicted in the painting differs from the rendering. However, the fact that the perceived differences are well explained by the differences in visual features confirms the role of these few visual features in material appearance.

#### Brightness rating

The paintings of nonmetallic materials were generally rated brighter, which is in accordance with the difference in *luminance* that we observed between renderings and paintings. We found a very high correlation (r=0.98) between the difference in ratings between paintings and renderings and the difference in *luminance*. This shows that the difference observed in the perception ratings can fully be explained by actual changes in the images.

However, this difference did not impact the perception of the other material attributes. This seems to validate the intuition of the painter that the luminance is not responsible for the perception of the material, at least in this special case, where one object is shown without context to set the luminosity of the scene.

#### Roughness rating

Although we observe very few significant effects of the interaction of materials and depictions for the *sharpness of reflections* rating and the matched spread of specular reflections α, bigger differences can be observed in the rating of the *roughness* attribute. The most specular materials ($M_{2,3}$ and $P_{3..5}$) were consistently rated as more rough in the paintings than in the renderings (particularly significant for $M_{2,3}$ and $P_{3}$). While the roughness is strongly linked to the sharpness of the reflections, it seems that participants were more sensitive to the specific visual features of paintings (such as brush strokes) when judging the roughness than when judging the other attributes related to the sharpness of reflections. The fact that this effect is visible only for the most specular materials suggests that the brush strokes had a particular effect on material that exhibits clear highlights, with these highlights exhibiting more texture in the paintings than in the renderings.

## Discussion

### Difference of perception between realistic renderings and paintings

By looking simultaneously at objective (extracted from images) and subjective (human judgments) features, we can better understand how users perceived the materials in the paintings. Although some of their answers were different between paintings and renderings, many of these differences can be explained by actual differences in image features that are known to be linked to material perception. This shows that other visual differences between the two modalities (such as brush strokes) only had a minor impact on the perception of material appearance.

As detailed in Influence of depiction and interactions with material, we found the most significant differences of answers between paintings and renderings for the *brightness* and *strength of reflections* attributes and for the matched contrast $\rho_{S}$. Our analysis shows that all these differences were due to actual differences in the stimuli: The difference in *luminance* explains well the difference in *brightness* rating while the difference in *high/low contrast* and *highlights sharpness* explains well the difference in all the answers that relate to the perception of specularities (*glossiness*, *strength of reflections*, and $\rho_{S}$). This shows that there is very little effect of the modality (renderings or paintings) on the perception of the low- and mid-level properties of the material, despite their important visual differences, such as the presence of brush strokes in the paintings.

Surprisingly, there are more differences in the answers of the classification, which represents a higher-level task. Users had a tendency to classify more materials as “Other” and “Fabric” when looking at the paintings than when looking at the renderings (mainly classified as “Metal” or “Plastic”), showing that the texture of the painting misled them. The full answers to the classification are shown in Appendix B.4 (Figure 16). Some users explained that they had the impression that all the paintings were representing some textured material, such as rocks. It thus seems that the brush strokes in the paintings were interpreted by some users as texture and influenced them in choosing categories that are more prone to present a textured surface. This could indicate that perceiving individual reflectance properties, such as glossiness or sharpness of reflections, is not sufficient to correctly identify the material and that the texture might play a bigger role for this task since the users were more sensitive to the painting “artifacts,” such as brush strokes, when trying to identify the material.

Additionally, it seems that users were not equally sensitive to the visual differences between paintings and renderings depending on the type of material properties they had to judge. We have seen almost no difference in answers for the perceptual attributes linked to the sharpness of reflections in both the rating and matching task. This could mean that users are less influenced by the artifacts of the paintings when judging the sharpness of highlights, which can be judged more locally than the glossiness or the strength of reflections. Interestingly, we observed a bigger effect of the medium on the *roughness* attribute, which is also supposed to be linked to the sharpness of reflections: Users rated the most glossy materials as rougher in the paintings than in the renderings. One should also consider that the term “roughness” can be ambiguous, since the resulting perceived roughness can be due to a combination of the artist's depiction of the material and the roughness of the painting medium itself, not present in the case of renderings. As such, even if the depicted material has a very small roughness (like $P_{5}$ or$M_{3}$), the painting medium brings some amount of roughness and prevents reaching a very low *roughness* score. This could explain the lower consistency that we observe between participants’ answers for this attribute on the paintings (average interparticipant correlation of 0.59): Some participants may have been more sensitive to the influence of the medium texture than others.

**Figure 15.**
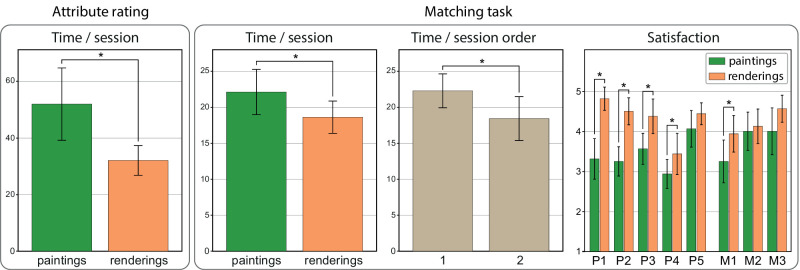
Time for completing the matching and ratings depending on the session (left) and block order (center). The right-most plot shows the satisfaction with the matching for each material according to the session (right). Error bars correspond to a 95% confidence interval. Asterisks indicate significance for the comparison between paintings and renderings for each material.

**Figure 16.**
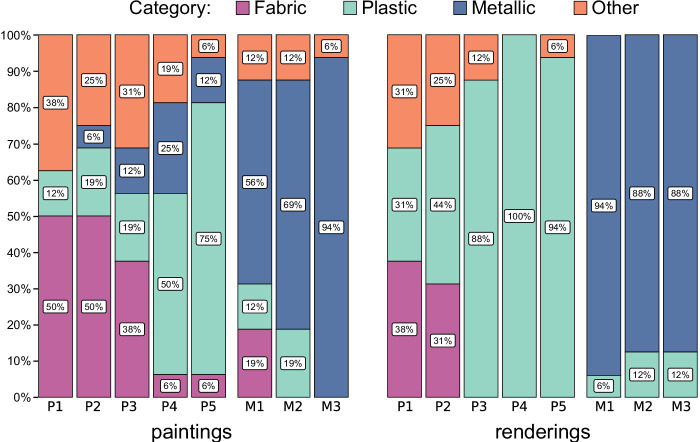
Number of assignment of the stimuli for each category of material.

Finally, all the tasks in the perceptual study were significantly longer to complete for paintings than for renderings, with the participants also being less satisfied with their matching for the paintings. The complete data are shown in Appendix B.3 (Figure 15). The difference in matching satisfaction could be partly explained by the fact that the reference image in the matching task is a rendered image. It is thus easier to match the renderings since they are visually more similar to the reference, especially when there is no strong highlights to use as a guide. However, the difference in time to complete the tasks cannot be explained in the same way, since the rating of the attributes was also significantly longer for the paintings. In the absence of context, the recognition of materials thus seems to be not as fast and natural in paintings as in natural images, as if users need some time to abstract from the painting artifacts and correctly judge the material properties. The fact that participants also seemed to have more difficulties judging the type of material that they were seeing, such as easily recognizing a plastic or a metal, could have played a role in this longer process to judge material properties.

### Depiction of material appearance

The results of the perceptual study show that the painter was successful in reproducing the appearance of the different materials, with the participants perceiving high-level attributes similarly between the paintings and the renderings. Although the paintings are not an exact copy of the renderings, the painter reproduced a number of visual features carefully, such as the intensity, the sharpness and rough placement of highlights, the contrast of the image, or the intensity of the shadows and reflections. He also intentionally did not reproduce some other features such as the luminance or the exact shape of each highlights, which he considered of less importance. This can indicate that the painter knew which image features are key to material appearance and was able to use them correctly.

By looking at how these image features can predict perceptual ratings, we can also identify which of these features are key to each specific reflectance property in each modality. Our findings fully align with what was previously shown in perceptual studies that only considered computer-generated images. We show in this study that the exact same image features are linked to the perception of material properties in realistic paintings, similar to the observations of Di Cicco et al. (2019). This suggests that, despite other visual differences between paintings and renderings, the reproduction of these key visual features by the painter was sufficient to provide a good appearance perception of the materials.

However, the paintings of the most diffuse materials ($P_{1...4}$) exhibit larger deviations of visual features and were perceived significantly differently in the paintings and in the renderings. These paintings exhibit exaggerated light effects that led users to consider them as more glossy or with a more important specular component than in the renderings. Although this could partly be due to the personal technique or ability of this particular painter, the painting medium itself also plays a role. The painting technique (which relies on superposing layers of strokes) makes it difficult to create perfectly smooth gradients but, on the contrary, favors the creation of sharp highlights with brush strokes. This was confirmed by the painter, who described the most reflective materials as “requiring more work” due to the complexity of the image, but considered the most diffuse materials to be the most complicated ones to depict correctly due to these subtle effects and transitions. Aside from the personal ability or technique of the painter, it is thus possible that such smooth surfaces are hard to reproduce with the particular painting medium but would be easier with others, like charcoal. Additionally, both the realistic style used in this study and the depicted object reinforced this difficulty: The painter attempted to faithfully reproduce the light gradients while he could have found ways to abstract them with a more abstracted style, and the depicted object exhibited very large smooth surfaces in comparison to the scale of the paint texture, making this problem more obvious.

## Conclusion

Our study reveals that, despite their visual difference, people perceive materials almost identically in realistic paintings as in renderings, even when no context is provided. This can be due to the fact that the painter faithfully reproduced the most important key features to material perception, such as the placement, contrast, and sharpness of highlights, as shown in previous studies. By intentionally reproducing these image features, the painter confirmed his implicit understanding of material perception. More important we show that these image features are linked to high-level perceptual properties in the same way in paintings and in renderings and that they can even be used to predict perceptual ratings. This suggests that our perception of materials relies mostly on a few key image features and that the reproduction of these features allows us to transmit material appearance. However, our study focuses on global statistics that can easily be measured by numbers but does not use other image features such as the placement of highlights or their alignment with the surface curvature, grazing angle effects, or any other light effects. Further research should be conducted in order to develop robust methods to measure or evaluate such features.

We also observed that the tasks were harder to complete for the paintings than for the renderings, showing that interpreting the material in paintings is not as natural as with natural images and requires some effort to abstract from the painting artifacts. Further studies could be conducted to better understand the process underlying this difference and study the influence of depiction style and the material. It is, for example, possible that the style of the painting plays a role or that some materials are easier to interpret in paintings.

Our study is currently limited to one medium and one style of painting. An extended study with various media (such as watercolor or pencil) involving different artists would be required to see if our findings hold in a more general context. Specifically, we focus here on realistic painting, with a high level of detail. A further study could also include different levels of abstraction to further understand up to which level of abstraction people are able to recognize material properties without context and if the image features that are reproduced are still the same. Additionally, the depicted object in this study exhibits a smooth surface, without small, high-frequency details in shape. Future studies could also include shapes with more geometric detail in order to see how painters depict the appearance of these intricate parts, whether they do it with an economy of means (see, e.g., the golden material in the inset in Figure 1a), and how the result of the more complex interaction between material and shape is conveyed.

## Figures and Tables

**Figure 11.**
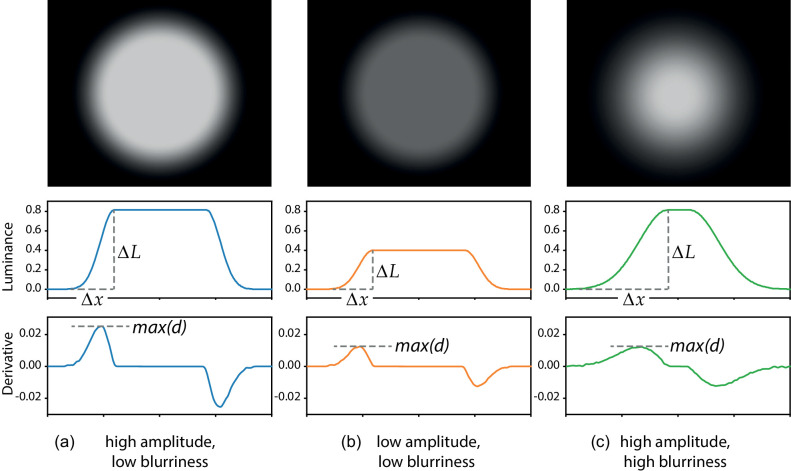
We define the *highlights amplitude* as ΔL and the *highlights blurriness* as Δx, the width of the transition. This last measure reflects well the visual sharpness at various contrast: (a) and (b) have a similar sharpness (small Δx) while (c) is blurrier (larger Δx). Using only the derivative biases results by contrast: (b) and (c) have the same derivative while they appear to have very different sharpness.

**Figure 12.**
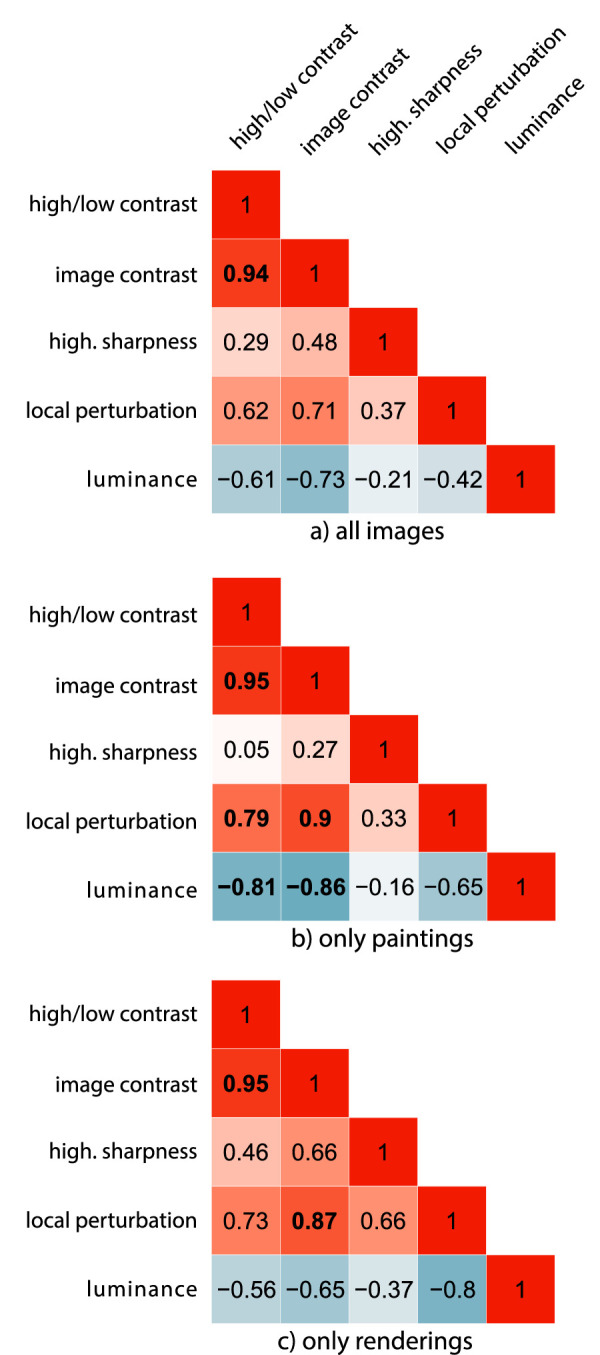
Correlations between our image features for (a) all the images, (b) only the paintings, and (c) only the renderings. The color scale indicates the strength and direction of the correlations (from blue to red). The most significant correlations (r>0.75 and p<0.001) are indicated in bold.

**Figure 14.**
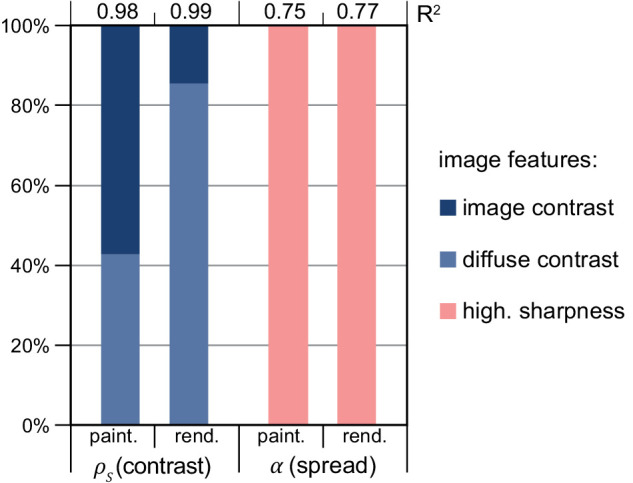
Coefficients in the linear models that predict each user's answers of the matching task. For each variable, the left bar shows the coefficients for the model fitted on the paintings while the right-most one shows the coefficients of the model fitted in renderings. The regression score for each model is displayed on top of each bar.

**Figure 17.**
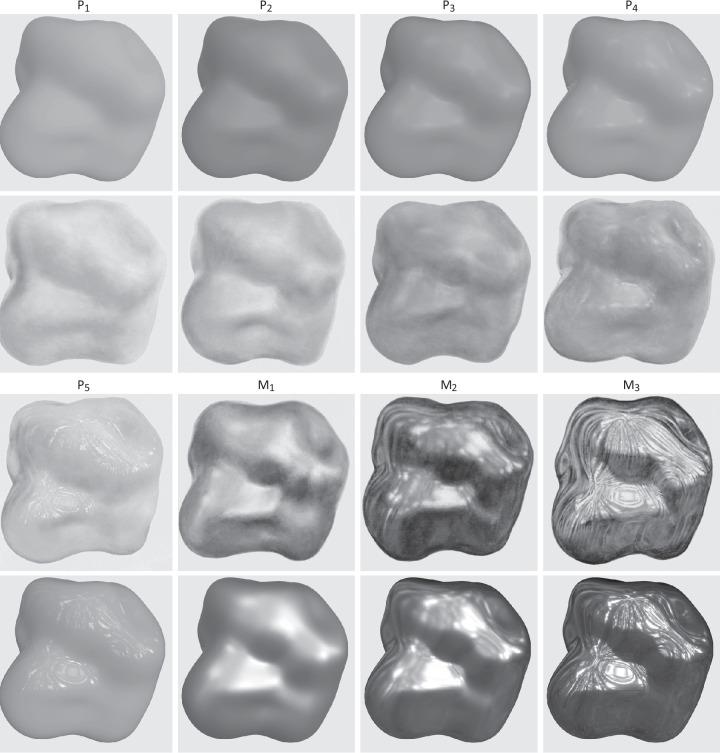
High-resolution version of the stimuli used in our perceptual study, including both renderings (first row of each pair) and paintings (bottom row), of each of the eight materials.

## Footnotes

1 Georges Ward.

## Appendix A: Computation of image features

### Computation of highlights sharpness

To measure the sharpness of the highlights, we take inspiration from Di Cicco et al. (2019) and use the width (measured in pixels) of the highlights transition rather than the derivative. This measure is not biased by the amplitude of the highlights, as shown in Figure 11. In this figure, the width of the transition is denoted as Δx while ΔL denotes the amplitude of the highlights. The first two images look similarly blurry despite the difference of amplitude; this is well captured by Δx, whereas the maximum derivative max(d) is different between the two images. On the other hand, the last two images have the same derivative but appear to have a very different sharpness, which is well captured by the width of the transition. In order to get a robust estimate of the derivative, we average the local maxima of the derivative if the profile contains peaks (derivative higher than a threshold) and we take the average derivative if no peaks are detected.

The computation actually give a measure of *blurriness* instead of a measure of *sharpness*. We thus reverse the measure and define our final *highlights sharpness* as 60-Δx.

### Correlations between the image features

We analyze how varied our image features are and we obtain their Spearman correlations, shown in Figure 12 for all the images, as well as for the paintings and renderings separately.

When considering all the images, our measures do not exhibit strong intercorrelations (>0.75) except for the two measures of contrast (*image contrast* and *diffuse contrast*) that are very highly correlated (r=0.94) since both are linked to the luminance amplitude in the image.

However, when looking at paintings and renderings separately, the *local perturbation* has strong correlations with both the *diffuse contrast* (r=0.79 and r=0.73, respectively) and the *image contrast* (r=0.9 and r=0.87, respectively). The *local noise* not only captures the brush strokes noise in the paintings but also partially accounts for the strength of the highlights and lowlights reflections, leading to a higher measure for the metallic materials, which correlates with the measures of contrast.

Finally, the other high correlations are caused by a bias in our selection of material. The correlation between the *luminance* and the *image contrast* (r=-0.73 for all images) is due to the fact that our selection of nonmetallic materials contains only luminous materials. This correlation is particularly high for paintings (r=-0.86) since the nonmetallic materials were painted brighter.

## Appendix B: Additional results of the user study

### Preliminary analysis: Correlations between user answers

#### Interparticipant correlations

We evaluate how consistent are the participants with each other by computing the pairwise Spearman rank correlations between each participants. We report in Table 1 the median of these pairwise correlations for each attribute or parameter of the perceptual study, for all images as well as for renderings and paintings separately.

**Table 1.** Median interuser agreement computed after pairwise Spearman correlations between our 16 users for the ratings and parameters of the matching task (all images, renderings, and paintings).

	All	Renderings	Paintings	
$\rho_{s}$ (contrast)	0.81	0.87	0.75	
α (spread)	0.91	0.96	0.88	
Glossiness	0.74	0.91	0.72	
Roughness	0.62	0.81	0.59	
Sharpness of reflections	0.78	0.81	0.82	
Strength of reflections	0.73	0.82	0.75	
Brightness	0.69	0.71	0.72	

We first analyze the answers when pooling all the images. Participants were the most consistent in their answers to the matching task, which is to be expected since there is a visual reference in this task. For the attribute rating, participant were consistent in their answers to *glossiness*, *strength of reflections* and *sharpness of reflections* (r>0.7). They were less consistent in their answers to *roughness* and *brightness*, showing that these attributes are more prone to personal interpretation and scaling. For the *brightness*, the answers of three participants have particularly low correlations with the answers of all the other participants, while the other participants were more consistent.

Globally, participants were less consistent in their answers for the paintings than for the renderings, showing that the paintings are more prone to personal interpretation. Additionally, the interparticipant correlations for the *roughness* attribute are low only for the paintings, which might indicate that participants were biased by the texture of the paintings when judging the roughness of the materials.

#### Interattribute correlations

In our experiment, we asked participants to rate various attributes that are known to be linked to each other (e.g., *glossiness* and *strength of reflections*) and to estimate similar properties through both the rating task and the matching task. We investigate if participants were consistent in their answers and if we retrieve the same relationship between attributes for both the paintings and the renderings. To do so, we compute the Pearson correlation between all the answers given in the experiments: the two matched values ($\rho_{S}$ and *α*) and the five rated attributes. The correlations are shown in Figure 13, for all the images as well as for the paintings and renderings separately. A blue color indicates a negative correlation while red indicates a positive correlation, with the saturation indicating the strength of the correlation.

We observe similar patterns of correlations for both the paintings and the renderings. The matched contrast $\rho_{S}$ and the rating of the *strength of reflections* are very highly correlated (r=0.87). This is to be expected since both measures are linked to the same reflectance property, answered within a different task (rating or matching task). Similarly, the matched α value and the rating of the *sharpness of reflections* are also very highly correlated (r=0.96). In addition, the *rough* attribute, which also relates to how sharp are the reflections, is highly correlated with these two answers (r>0.95). This shows that participants were consistent in the way they perceive the sharpness of the reflections.

Finally, the rating of the *glossy* attribute is highly correlated with both the *strength of reflections* (r=-0.79) and all the measures that relate to the sharpness of the reflections (r>0.9). This is in accordance with several studies that have shown that glossiness depends on both the contrast and the sharpness of highlights (Pellacini et al., 2000; Marlow & Anderson, 2013; Di Cicco et al., 2019).

These results show that participants were able to answer consistently for renderings and paintings, both for the low-level representation (matching task) and for the high-level perceptual properties (rating task).

### Fittings of the linear models to matched parameters

Figure 14 shows the linear coefficient and regression scores of the linear models fitted to the answers of the matching task. Similarly to the linear models fitted on the answers to the rating task, the coefficients and regression score are very similar between the paintings and renderings. The only difference lies in the prediction of the matched contrast $\rho_{S}$, which depends almost only on the *high/low contrast* for the renderings while it mainly depends on the *image contrast* for the paintings. This might be due to an important difference between the *high/low contrast* and the *image contrast* in the painting of $M_{3}$: The dark areas of the shape were painted much brighter than the original rendering, leading to an important loss of *high/low contrast*, but had less impact on the *image contrast* and on the perceived properties.

### Effects of the session on time and satisfaction

Both tasks were faster to complete for the renderings than for the paintings, as shown in Figure 15. Additionally, the users were consistently more satisfied with their matchings for the renderings than for the paintings. This effect was particularly observed for rougher materials: $M_{1}$ and $P_{1..4}$ (Figure 15, fourth column). Paintings of rough materials might be harder to match because the effect of the brush strokes is more important than in the specular ones.

This might be due to paintings being harder to interpret out of context, requiring some time and effort to judge the material properties. Moreover, since the reference image in the matching task is a rendered image, it may be easier for participants to match the renderings, since they are visually more similar to the reference.

We also have found a significant influence of the block order in completion time of the matching (Figure 15, third column). In particular, the second block carried out was consistently completed faster. We believe that users got used to the slider interface in the first block and therefore were able to complete the task faster in the second block. Note, however, that this did not have an effect in the final performance of the matching. Finally, we did not find any significant effect of the order in which the two blocks were carried (paintings or renderings first) on any of the parameters or attributes.

### Material classification

Figure 16 shows the choices of the different material categories for each material, in the paintings and in the renderings.

In general, the metallic materials $M_{1..3}$ were classified as “Metallic” the majority of the time while other materials were classified either as “Fabric” or “Other” (for the most diffuse ones) or as “Plastic” (for the most glossy ones).

When looking only at the paintings, this classification is less clear. For the materials that have very defined reflections ($M_{3}$ and $P_{5}$), the answers are similar between the two modalities. Among the other materials, the metals were less classified as “Metal” in the paintings while $P_{1..4}$ were less classified as “Plastic,” with some of them even being classified as “Metal.” Globally, the paintings were more often viewed as representing “Fabric” or an “Other” material. Some of the users reported that they had the feeling that all the paintings were representing some kind of textured material such as “rocks” or “clay.”

We also analyzed the link between our image features and these classification answers by computing their point-biserial correlations. Most of the correlations that we observed are small (under or around 0.5), indicating that material classification cannot be easily linked to individual low-level image features. However, we observed relatively strong correlations (around or above 0.7) between both the *high/low contrast* and the *image contrast* and the Metallic classification. This is in accordance with what we observed in our image features: Metallic materials have much higher contrasts due to their dominant specular component. Further investigation would be needed to understand the role of the different image clues and low-level perceptual properties on the material classification.

### Influence of demographics on user study

#### High-level attribute rating

Computer graphics (CG) experience had a significant influence only in the ratings for glossiness (*F*(3, 233) = 8.899, *p* < 0.001) and brightness (*F*(3, 233) = 8.965, *p* < 0.001). Post hoc analyses revealed that in both cases, users with no experience rated those two attributes consistently lower. Art experience only had a significant effect on the glossiness attribute (*F*(2, 233) = 3.725, *p* = 0.026), with users with intermediate art experience rating glossiness higher.

#### Matching task

We have found a significant influence of the experience on CG for both parameters of the model, as well as the satisfaction and the time to completion. The art experience was only significant for the parameter alpha. For the contrast, users with less CG experience in general matched lower values than users with intermediate or professional experience (*F*(3, 233) = 4.397, *p* = 0.005). For the spreading of the specular reflection α, both for CG experience (*F*(3, 233) = 370.456, *p* < 0.001) and art experience (*F*(2, 233) = 4.740, *p* = 0.01), users with less experience tended to match to higher values. For the satisfaction and time to complete the match, users with less CG experience tended to be less satisfied with their results (*F*(3, 233) = 10.198, *p* < 0.001) but also spent less time performing the matching (*F*(3, 233) = 13.808, *p* < 0.001).

## Appendix C: High-resolution stimuli

In Figure 17, we show the stimuli (paintings and renderings) in high resolution, following the same ordering as in the main text (Figure 2).
